# Large-scale automated machine reading discovers new cancer-driving mechanisms

**DOI:** 10.1093/database/bay098

**Published:** 2018-09-26

**Authors:** Marco A Valenzuela-Escárcega, Özgün Babur, Gus Hahn-Powell, Dane Bell, Thomas Hicks, Enrique Noriega-Atala, Xia Wang, Mihai Surdeanu, Emek Demir, Clayton T Morrison

**Affiliations:** 1 Department of Computer Science, University of Arizona, Tucson, AZ, USA; 2 School of Medicine, Oregon Health & Science University, Portland, OR, USA; 3 Department of Linguistics, University of Arizona, Tucson, AZ, USA; 4 School of Information, University of Arizona, Tucson, AZ, USA; 5 Department of Molecular and Cellular Biology, University of Arizona, Tucson, AZ, USA

## Abstract

PubMed, a repository and search engine for biomedical literature, now indexes >1 million articles each year. This exceeds the processing capacity of human domain experts, limiting our ability to truly understand many diseases. We present Reach, a system for automated, large-scale machine reading of biomedical papers that can extract mechanistic descriptions of biological processes with relatively high precision at high throughput. We demonstrate that combining the extracted pathway fragments with existing biological data analysis algorithms that rely on curated models helps identify and explain a large number of previously unidentified mutually exclusive altered signaling pathways in seven different cancer types. This work shows that combining human-curated ‘big mechanisms’ with extracted ‘big data’ can lead to a causal, predictive understanding of cellular processes and unlock important downstream applications.

## Introduction

**Figure 1:**
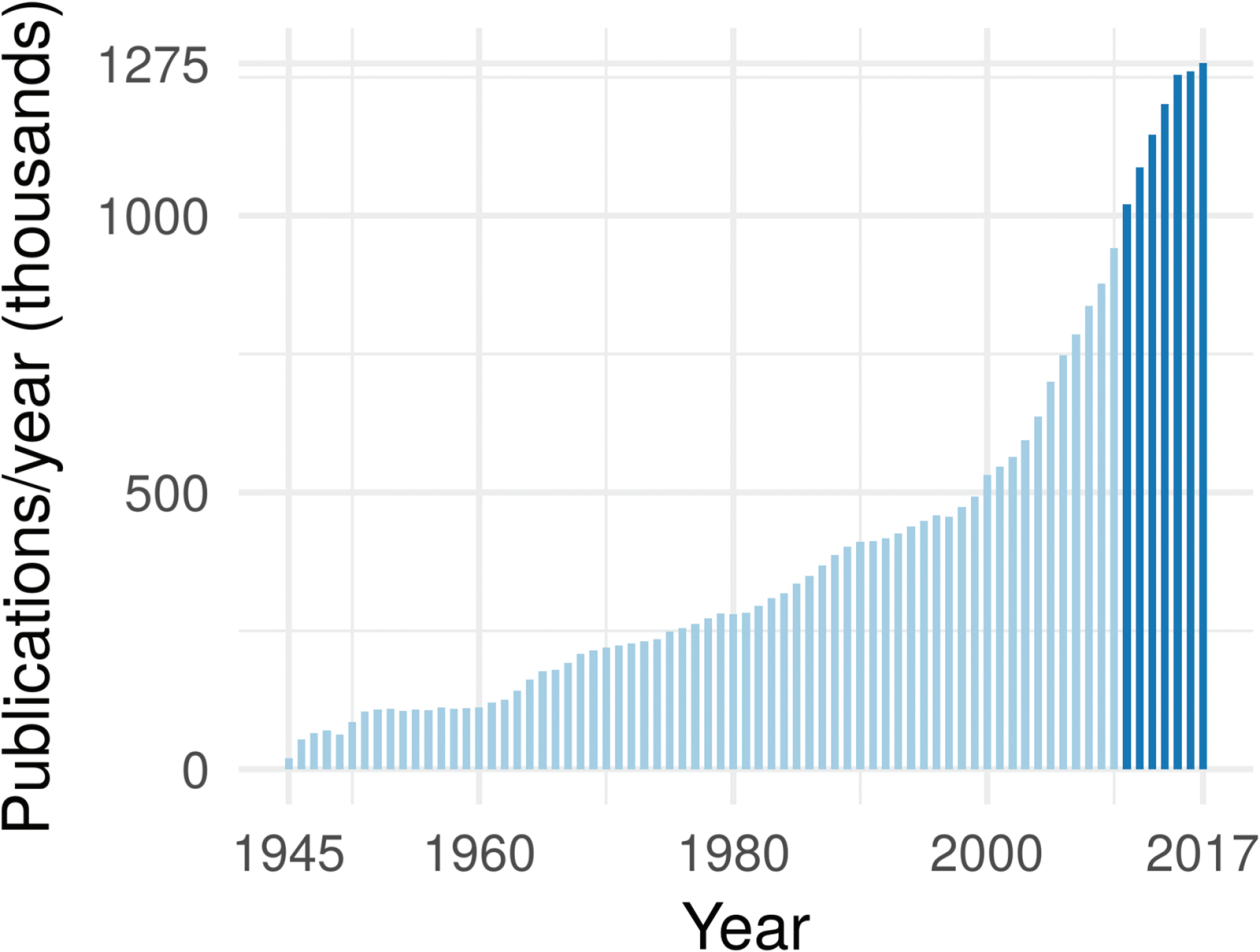
The annual rate of publications in the biomedical domain, as indexed by PubMed. The darker blue highlights that publications have exceeded 1 million per year starting in 2011.

In the past 7 years, >1 million publications were added to PubMed each year (43) (see Figure 1). At the same time, a typical large-scale patient profiling effort now produces petabyte of data and is expected to reach exabytes within the near future (38). Combining these large data sets with mechanistic biological information can lead to a causal, predictive understanding of cellular processes and can unlock important downstream applications in medicine and biology.

Unfortunately, most of the mechanistic knowledge in the literature is not in a computable form and mostly remains hidden. Existing biocuration efforts are extremely valuable for solving this problem, but, unfortunately, they are out-scaled by the explosive growth of the literature. For example, we estimate that public pathway databases such as Pathway Commons (PCs; www.pathwaycommons.org) capture only 1–3% of the literature and the gap widens everyday (internal analysis of the PCs team).

This gap severely limits the value of big data in biology. As a concrete example, consider the detection of ‘driver’ mutations in cancer. One widely recognized observation is that, given a cohort of patients, some driver alterations will exhibit a mutually exclusive (or *mutex*) pattern. That is, the number of patients that have both alterations will be smaller than what is expected by chance. This often happens because these alterations unlock the *same* cancer driving pathways and the positive selection of one diminishes substantially when the other is present. In other words, ‘one is enough.’ Prior pathway knowledge can be used to improve the accuracy of these methods by limiting the search space and reducing the loss of statistical power due to multiple hypothesis testing correction. It also provides mechanistic explanations of the observed correlations (5). Recall, however, can be low because of the aforementioned database coverage issues. Researchers are thus faced with a choice between no-prior, high-coverage methods that do not provide mechanistic explanations or low-coverage, prior-based methods that may overlook some key events.

To fully answer such complex biological questions, we propose a natural language processing (NLP) approach that captures a system-scale, mechanistic understanding of cellular processes through automated, large-scale reading of scientific literature and demonstrate that this approach leads to the discovery of novel biological hypotheses for multiple cancers. We call our approach Reach (REading and Assembling Contextual and Holistic mechanisms from text).

Our approach has two important contributions. The first contribution is the demonstration that the combination of ‘big data’ that are produced by machines and ‘big mechanisms’ that were manually curated yields novel knowledge that is otherwise missed. In particular, we show that Reach can substantially improve the inference capacity of existing biological data analysis algorithms that previously relied solely on manually curated pathway databases such as PCs. Here, we extended the PCs human-curated pathways with >1 million biochemical interactions extracted by Reach from all papers in the Open Access subset of PubMed (as of June 2015). Using this combined prior network we were able to identify a large number of previously unidentified but highly statistically significant mutually exclusively altered signaling modules in TCGA (The Cancer Genome Atlas; https://cancergenome.nih.gov/) cancer data sets using the Mutex algorithm (5). A manual evaluation of these modules reveals that between 65 and 80% of the pathway fragments discovered by Reach are correct, and they indeed help elucidate novel biological hypotheses within the corresponding cancer context.

Our second contribution is the machine reading approach itself. The core of Reach is a cascade of automata that relies on *compact* and *interpretable* grammars that extract entities (e.g. proteins) and events (e.g. biochemical interactions) of interest. This guarantees that the reading model can be understood, modified and extended by domain experts. This compact grammar is efficiently applied at runtime, an important benefit in our ‘big data’ setup. On average, we process a single paper in 4.5 seconds, though our software can be easily parallelized to take advantage of cluster computing and multi-core hardware. Additionally, Reach captures complex natural language phenomena such as coreference and can interpret event polarity in statements with nested contrasts (for example, ‘a *reduction* of *increased* phosphorylation led to …’). An independently administered evaluation found that Reach extracts cancer signaling pathways at relatively high precision and at a throughput capable of reading the whole biological literature in short order.

The inherent inter-disciplinarity of this work has yielded an unorthodox paper structure. We dedicate the first half of the paper to the introduction of our machine reading approach and its intrinsic evaluation (2 and 3). The second half of the paper then provides a brief summary of the biological data analysis algorithm used in this work (4), followed by an extrinsic evaluation of machine reading, which measures the contribution of machine reading to the detection of novel biological hypotheses (5).

## Machine reading approach

**Figure 2:**
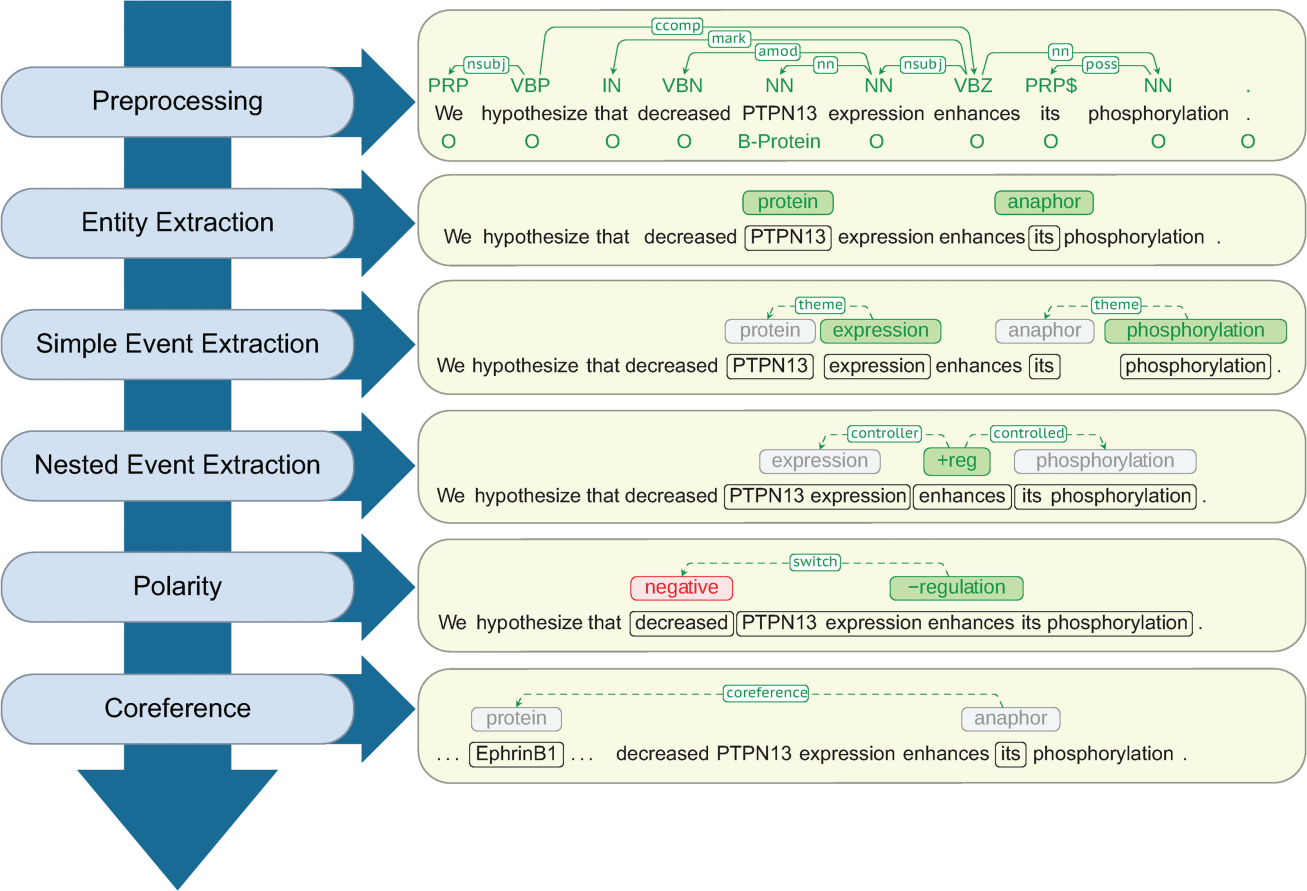
Architecture of the Reach system together with a walk-through example.

At a high level, Reach uses a cascade of rule-based and statistical techniques to read the content of a paper and identify mentions of molecular events that describe fragments of a signaling pathway.The steps of this sequence, as shown in Figure 2, proceed from low- to high-complexity representations, each building on the output of the previous steps.

The representation of these mentions is constructed internally in a format inspired by the BioPAX standard language (16). Notably, Reach can represent detailed biochemical conversions where entities go through ‘state’ changes, such as becoming phosphorylated or changing their sub-cellular location. Reach also represents controllers or catalysts of these conversions when they are mentioned in text. Similar to BioPAX, these mentions are represented using a *composite* structure where events can have other events as their participants, allowing for arbitrarily complex logic. An important extension of BioPAX that Reach implements is the extraction of higher-level control relations between entities (e.g. ‘KRAS activates p53’). Although such relations are biologically ambiguous relative to a mechanistic conversion representation (e.g. the above example summarizes the biological mechanism ‘KRAS promotes phosphorylation of p53 on Ser37’), they provide valuable information to domain experts.

In the following sections we describe details of the Reach architecture components in Figure 2 that are responsible for the extraction of these mechanism fragments.

### Preprocessing

Reach first preprocesses the text with NLP tools specifically modified for the biomedical domain. Preprocessing includes sentence and word segmentation, part-of-speech (POS) tagging and syntactic parsing.

The sentence and word segmentation step detects both sentence and word boundaries in the input text. There are subtle but important differences between the tokenization of open-domain text and biomedical content. For example, dashes that occur within a word are not considered separators when segmenting open-domain text, but they tend to function as word separators in biomedical texts. For example, segmenting the text ‘GAP-mediated’ at the dash is crucial for the downstream components to understand that this text contains a catalysis driven by GAP. Similarly, not considering the dash as a separator would prohibit the downstream components from recognizing members of protein complexes, which typically appear as dash separated in text. To handle these phenomena, a custom segmenter was developed in-house, following the tokenization specification of the GENIA corpus (21).

For POS tagging and syntactic parsing, Reach uses Stanford’s CoreNLP toolkit (28), which has been trained using a combination of two corpora: the Penn Treebank, a corpus that merges several non-biomedical genres such as IBM computer manuals and Wall Street Journal articles (29, 39), and the GENIA corpus, which is a manually annotated corpus of 2000 MEDLINE abstracts (21). Including the GENIA-annotated documents as part of the parser’s training corpus makes the parser more robust to syntactic structures often found in biomedical literature.

### Entity extraction

**Figure 3:**
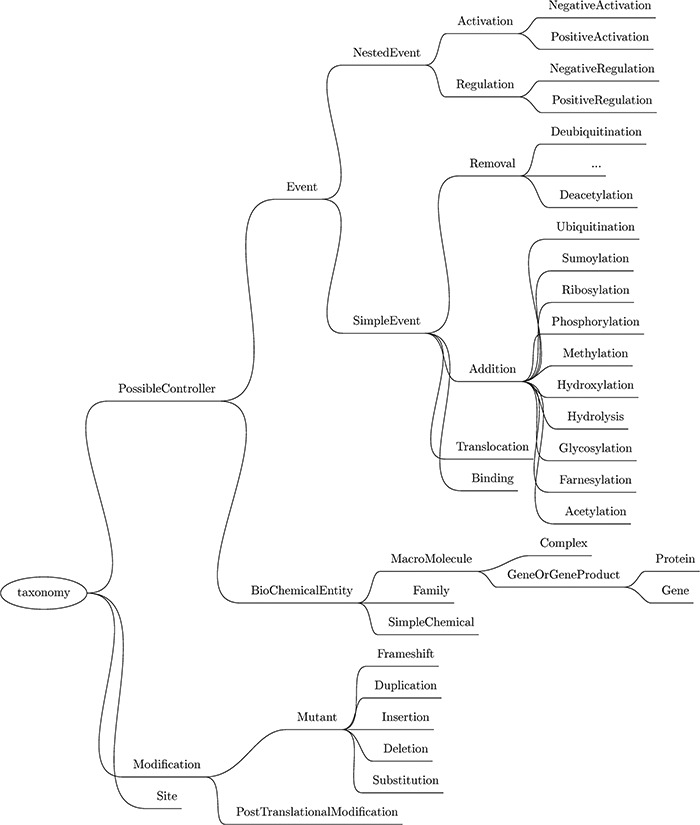
Taxonomy of the entities and events recognized by Reach. Though abbreviated, the Removal events mirror those listed under Addition.

Next, a custom named entity recognizer (NER) component is used to recognize mentions of relevant physical entities by type, such as protein family, cellular component, simple chemical, site and gene or gene product (this last category includes genes and proteins). The complete list of entities recognized by Reach, as well as the biochemical events described later, is listed in the taxonomy in Figure 3.

The custom NER uses a hybrid approach that combines a rule-based component with a statistical one. The rule-based component recognizes all mentions of known entity names (and their synonyms) from the knowledge bases (KBs) shown in Table 1. Additional grammar rules were written to capture entities that are not adequately covered by these KBs, such as cellular components or sites of biochemical reactions. The statistical model is implemented using CoreNLP’s conditional random field (CRF) sequence classifier, trained on the BioCreative corpus (19). This data set supports only mentions of gene or gene products. The hybrid NER combines the output of the two components, prioritizing the rule-based component when overlaps are detected.

**Table 1:** KBs used by the rule-based NER, as well as for grounding

*Entity Type*	*Database*	*URL*
Protein	UniProt	www.uniprot.org/
Protein families	InterPro	www.ebi.ac.uk/interpro/
Simple chemicals	HMDB	www.hmdb.ca/
Simple chemicals	ChEBI	www.ebi.ac.uk/chebi/
Sites	InterPro	www.ebi.ac.uk/interpro/

Next, Reach ‘grounds’ the biochemical entities discovered by linking the textual mentions to IDs of actual entities in the KBs shown in Table 1. For example, the protein mention ‘MEK1’ can be linked to the ID Q02750 in the Uniprot KB.

Lastly, Reach detects mentions of gene mutations and protein post-translational modifications (PTMs) and attaches them to the corresponding textual mentions of these biochemical entities. This is implemented with a subsequent grammar that focuses on detecting changes of states in the previously extracted entity mentions, e.g. from the text ‘wild type EHR’ Reach extracts the state Wild Type (i.e. non mutated) and attached it to the previously extracted entity mention ‘EHR’. (We will extend this component in future work to include binding sites and fragments.)

### Event extraction

Once Reach has determined which entities are mentioned in the text, it extracts the biochemical processes in which they participate. We use a two-step bottom-up strategy for event extraction, following biochemical semantics inspired by BioPAX. First, we identify biochemical reactions that operate directly on entities, temporarily ignoring their catalysts and other controllers (e.g. phosphorylation of a protein). Following NLP terminology, we call these events ‘simple’. Second, we find the processes that control these conversions (e.g. the catalysis of this phosphorylation by a kinase). We call these events ‘nested’, because of the fact that they have other events as their targets (e.g. the above catalysis operates on a phosphorylation simple event).

**Table 2:** Common syntactic variations shared among event types. Combinations of these syntactic variations are also considered. For example, an appositive subject relative plus passivization: ‘Pde2, which has been found to hydrolyze Ras, activates MEK’

*Name*	*Description*	*Example*
Declarative	The theme (the thing acted on by the verb) is the direct object of a verb.	‘Smurf1 and Smurf2 degrade and ubiquitinate RhoA.’
Passive	The theme is the syntactic subject of a verb phrase.	‘RhoA is ubiquitinated and degraded by Smurf1 and Smurf2.’
Prepositional nominalization	The trigger is in noun form and entities are in prepositional phrases.	‘The ubiquitination and degradation of RhoA by Smurf1 and Smurf2 increased.’
Object nominalization	The trigger is in noun form and with the theme forms a noun-noun compound.	‘RhoA ubiquitination and degradation by Smurf1 and Smurf2 increased.’
Subject nominalization	The trigger is in noun form and with the cause forms a noun-noun compound.	‘Smurf1 ubiquitination and degradation of RhoA increased.’
Subject relative clause (+ optional apposition)	The trigger and theme are located in a relative clause which modifies the cause.	‘Its many abnormal phenotypes can be rescued via Pde2, which specifically hydrolyzes cAMP.’
Object relative clause (+ optional apposition)	The trigger and cause are located in a relative clause which modifies the theme.	‘We measured transcription activation in the presence of cAMP, which is hydrolyzed by CRP.’
Subject apposition	The cause is in an appositive phrase.	‘Via yeast two-hybrid screening, we found that a novel protein, A20, binds to ABIN.’
Object apposition	The theme is in an appositive phrase.	‘Via yeast two-hybrid screening, we found that A20 binds to a novel protein, ABIN’
Paraphrastic causative	The trigger is separated from an entity by a verb.	‘Smurf1 causes the degradation of RhoA.’

One notable contribution of this work is the small number of rules used for event extraction. This is achieved by first identifying several general syntactic variations shared among event mentions and then reusing the same syntactic structures for all event types. Table 2 describes 10 syntactic variations used in this work, together with examples for each.

We implement the above intuition using templates expressed in the Odin information extraction rule language (40,42). Odin templates enable expression of rules representing parameterized patterns. For example, we used one template to describe the declarative syntactic pattern in Table 2, but left the actual verb as a parameter to be instantiated later. The particular verb to be used at runtime is initialized with specific values for the different event types (e.g. ‘phosphorylate’ for phosphorylation events).

In all, we support 12 different types of simple events, as highlighted in Figure 3. Nine of these are biochemical reactions: phosphorylation, ubiquitination, hydroxylation, sumoylation, glycosylation, acetylation, farnesylation, ribosylation and methylation. All of these reactions involve the covalent modification of a protein. The difference between these events and the PTMs extracted in the previous step is that these events refer to the actual act of modifying the protein by attaching a functional group to it, and the PTMs described in the previous step refer to proteins that have already been modified (potentially as a result of simple events mentioned previously in the paper).

The three remaining simple events are translocation, which refers to the act of transporting an entity between two cellular locations; binding, which is the process of assembling a complex from two or more proteins; and hydrolysis, the separation of chemical bonds by the addition of water. Hydrolysis captures activities like cleavage and degradation.

Nested events are processes that control other events, such as catalysis and inhibition. Reach recognizes both positive (e.g. ‘promotes’) and negative (e.g. ‘inhibits’) controls. It is also possible to chain the control logic, e.g. the co-modulation of a catalysis. Following BioNLP terminology (22), we collectively call these types of events ‘regulations’ for simplicity.

Reach also recognizes mentions of ‘activations’, i.e. higher-level interactions that describe the direct control of an entity’s activity (e.g. ‘A activates B’, where A and B are proteins). These are structurally very similar to regulations with the exception that the ‘controlled’ participant is an implied downstream activity of a biochemical entity. These are not supported in BioPAX by design because of the inherent semantic ambiguity: proteins can have multiple, overlapping ‘activities’. Reach supports them because they are abstractions frequently used to summarize the result of a sequence of steps in a signaling pathway. These activations are not as useful as regulations when considered in isolation, but they provide valuable information, including the author’s high-level interpretation of the discussed mechanism and indirect dependencies between proteins. In the next section, we demonstrate how to use this information to discover latent explanations for cancer drivers.

Similar to simple events, nested events conform to the syntactic patterns shown in Table 2. Capitalizing on these patterns, the extraction system was implemented in Odin using 154 unique rule templates, as shown in Table 3.

**Table 3:** Number of rule templates in Reach’s grammars

*Type*	*Syntax*	*Surface*	*Total*
Entities	0	15	15
Generic entities	0	2	2
Modifications	0	6	6
Mutants	0	9	9
*Total*	0	32	32
Simple events	15	11	26
Binding	30	7	37
Hydrolysis	8	2	10
Translocation	12	0	12
Positive regulation/activation	16	4	20
Negative regulation/activation	14	3	17
*Total events*	95	27	122
*Total*	95	59	154

### Complex natural language phenomena

In addition to the event and entity extraction grammars described previously, Reach also recognizes complex phenomena that are difficult to detect with rules alone, namely polarity and coreference.

**Polarity.** Special treatment is needed for statements that involve nested controls with different polarities. For example, in the text from Figure 2, ‘decreased PTPN13 expression enhances EphrinB1 phosphorylation’, the predicate ‘enhances’ seems to indicate that PTPN13 up-regulates the phosphorylation of EphrinB1. A careful inspection of the context reveals that it is the ‘decrease’ of PTPN13 that enhances the phosphorylation. This is interpreted by Reach as a polarity flip for the regulation of the phosphorylation (from positive to negative).

We handle polarity correction by traversing the syntactic dependency path that connects the trigger of the corresponding event and all its arguments in the syntactic dependency graph, keeping track of polarity-reversal words. Adjectival modifiers that connect to the path at any point are also considered. For example, in the regulation event depicted in Figure 2, the adjectival modifier ‘decreased’ signals the polarity reversal.

**Coreference resolution.** Coreference, the ability for different mentions in text to refer to the same real-world entity or event, is common in the biomedical domain. Resolving these coreference links leads to greater recall in information extraction, but it is rarely pursued in the biomedical domain. Coreference applies to both entities and events and often reaches across sentence boundaries, as in the following examples, in which the **bold text** refers back to the *italicized text*. The correct coreference resolution in each case allows a further event to be extracted.

- ‘In the current study, we describe the phosphorylation, localization and genome-wide regulatory functions of *HP1$\gamma $* in gonadal tissue, gametes, and the pre-implantation embryo. We demonstrate that phosphorylation of **this protein** at S83, which occurs in response to Aurora A, is necessary for supporting proper mitotic cell division in cells from the sperm lineage.’
- ‘When Wnt signaling or Cdc42 activity was blocked, the induced, but not the basal level of this interaction, was lost, suggesting both Wnt and Cdc42 activities are required to promote a *Dvl2/aPKC interaction* after scratching. In contrast, aPKC inhibitors did not block **this interaction**, suggesting aPKC activity was not required for Dvl2/aPKC complex formation.’

Inspired by Lee *et al.* (27), we adopted an architecture for resolving coreference in which deterministic resolution rules (or ‘sieves’) are ordered from highest to lowest precision and from lowest to highest recall. The advantages of this approach are similar to those of the previously introduced rule-based architecture for entity and event extraction, including stability, human interpretability and high overall performance.

However, though successful in the open domain, we discovered that the system proposed by Lee *et al.* (27) is not well suited to the biomedical domain, producing low-precision results due to over-clustering. To account for this, we adapted the sieves to the biomedical domain by eliminating sieves that are redundant, uninformative in this domain or insufficiently restrictive, as well as by creating new, domain-specific sieves that capitalize on domain knowledge.

For example, recognizing mutants (though the word *mutant* may not appear) will allow linking in sentences such as ‘…we prepared recombinant *H2AX-K134A*…The intensity of the band corresponding to histone H2AX methylation was significantly diminished in **the K134A mutant** compared with that of wild-type H2AX (H2AX-WT)….’. Similarly, recognizing specific protein reactions allows otherwise difficult resolution, as in linking two dissimilar mentions of a single binding reaction in ‘*LL-37 forms a complex together with the IGF-1R* …and **this binding** results in IGF-1R activation ….’ We described this approach in detail in (8).

## Intrinsic evaluation: machine reading performance

### Comparison with other reading systems

In an independently administered evaluation (36) [conducted by MITRE in the DARPA Big Mechanism program (www.darpa.mil/program/big-mechanism)], Reach was found to extract signaling pathways at relatively high precision, at a throughput capable of reading the entire open source biomedical literature within days. Participating systems extracted mechanistic information from 1000 papers about the Ras signaling pathway over the course of a week. Two metrics were used to evaluate the participating systems: (i) *precision*, calculated as the proportion of interactions that were considered ‘largely correct’, i.e. (a) the interaction had to match the text evidence, (b) both participants (if present in the interaction) as well as the interaction type had to be correct and (c) the negative information indicator (was the interaction negated or not in text?), had to be correct; and (2) *throughput*, the estimated number of ‘largely correct’ interactions produced from the 1000 publications per day. Note that the correctness of entity grounding (i.e. linking the textual mentions of interaction participants to ids of actual entities in KBs) was not a factor in calculating this precision measure. Further, in this evaluation, throughput was used as a proxy for recall because true recall would be expensive to compute on such a large data set.

Four other teams participated in the evaluation. The participating teams followed different approaches. For anonymity, we do not identify the participating consortia by name, but briefly describe their approaches. Team 1 implemented a pipeline of machine learning components that addressed various aspects of the task, such as identifying interaction types, interaction participants etc. Teams 2 and 3 implemented a hybrid approach, where they used machine learning to construct semantic representations of the text (2, 7) and a rule-based component to extract domain-specific information from this open-domain semantic representation. Team 4 used a rule-based approach, with rules that focused solely on surface patterns. In this evaluation, Reach and Team 4 were part of the same consortium and evaluated jointly. The results are summarized in Table 4. (Please note that the precision scores in the table are based on a slightly different composition of papers for each team. The reason MITRE did this is that the number of interactions generated varied greatly among teams; the evaluation team had to score interactions from more papers to get reasonable precision numbers for submissions with fewer extractions. In particular, all participants were scored on outputs from the same eight papers; but Team 1, Team 3 and Reach + Team 4 were evaluated on two additional publications, and Team 3 was further evaluated on three more.)

**Table 4:** Machine reading results in the Big Mechanism evaluation

*Team*	*Throughput*	*Precision*(%)
Team 1	62	59
Team 2	342	23
Team 3	110	**63**
Reach + Team 4	**695**	49
Reach	486	62

The table shows that the Reach + Team 4 consortium obtains the best balance of precision and throughput, with the highest throughput and relatively high precision. Team 2 had the next highest throughput, but both its throughput and precision were more than twice as low as Reach + Team 4’s scores. Teams 1 and 3 had higher precision scores, but their throughputs were considerably smaller: 11.2 and 6.3 times smaller than Reach + Team 4’s throughput, respectively.

While this evaluation reports results for Reach and Team 4 jointly, we aimed to tease out Reach’s contribution in this consortium. To this end, we performed a post-hoc internal analysis of the data generated for this submission, separating the extractions produced by Reach from the extractions produced by Team 4. This analysis showed that Reach alone has a precision of 62% and is responsible for $\sim\!70\%$ of the consortium throughput. These results, shown in the last row of Table 4, support the same observations: Reach has a throughput considerably higher than all the other teams, at precision approaching the highest precision value observed in the evaluation.

The high throughput observed for Reach has two causes. First, the approach implemented in Reach, which includes Odin grammars that cover both syntax and surface patterns, coreference resolution, polarity handling etc. (see previous section), guarantees good coverage of the various linguistic phenomena encountered in this data. Second, the Reach grammar runtime system is fast: on average Reach processes a publication in <5 seconds. This allowed the team to easily process the entire data set of 1000 papers in the time allotted for this evaluation. In fact, the Reach submission was completed in the first few hours of the first of the 7 days reserved for the evaluation.

All in all, this analysis demonstrates that Reach manages to maintain comparatively high precision without considerably sacrificing throughput. As we show in 5, this high throughput can be leveraged to increase precision by taking advantage of redundancy, i.e. the more times an interaction is extracted, the more likely it is to be correct.

### Other biomedical tasks

Note that, while other efforts on extracting biomedical structures from free text certainly exist (22–24, inter alia), they are not directly comparable to this work, for several reasons:

1. There are differences in task definitions between Big Mechanism and other existing efforts. For example, the events covered in the BioNLP data sets (22–24) include gene expression and transcription interactions, whereas Reach focuses strictly on post-translational modification (PTM) events. On the other hand, the BioNLP data sets focus on molecular-level regulation events, whereas Reach additionally extracts activation events that describe interactions at a higher abstraction level. Furthermore, there are differences in how interactions were defined in BioNLP vs. Big Mechanism. For example, Binding (i.e. complex assembly) events in BioNLP can have an arbitrary number of arguments, whereas in Big Mechanism Binding events are binary ($n$-ary complex assembly interactions are represented as a sequence of binary Binding events).
2. There are considerable tokenization differences between BioNLP annotations and Reach. Specifically, BioNLP extracts subword events, e.g. where both the predicate and the corresponding argument are included in the same token, and subword arguments, e.g. where only part of a word is the argument of a predicate. For example, ‘phospho-p38’ is labeled as an event in which the p38 protein is phosphorylated. Reach generally does not extract such subword events.
3. Most BioNLP data sets contain only text from publication abstracts (22), or a mixture of abstracts and full publications, heavily biased toward abstracts (23). The BioNLP 2013 data set (24) is the only one that contains solely text from full publications, but it is small (only 10 publications), which introduces a bias risk. In contrast, Reach was designed to robustly parse the full content of any biomedical paper.

Nevertheless, in order to put this work in a larger context, we implemented a simple comparative analysis in which we evaluated Reach on Phosphorylation events in the BioNLP 2013 data set. Phosphorylation interactions are the most frequent PTM simple event in the BioNLP 2013 data set, and they generally align well with the Reach definition. On the BioNLP 2013 development partition, Reach obtains a precision of 92.9%, a recall of 56.0% and an F1 score of 69.9% (using the approximate span and recursive criteria scorer, the standard scorer configuration in the BioNLP challenge). In contrast, the Turku Event Extraction System (TEES) (9), the second best system in the BioNLP 2013 evaluation, obtained 83.9% precision and 83.5% recall on the development data set. We find Reach’s high precision encouraging, especially considering that Reach was never exposed to this data set before this exercise, whereas all the other BioNLP participants used supervised learning and tuned hyper parameters to maximize performance on this development partition.

To understand the lower recall, we inspected the false negatives (FNs), i.e. phosphorylation events missed by Reach on the development partition of the BioNLP 2013 data set. Our analysis confirms that these were caused by differences in task definition. In particular, 58% of the FNs were caused by subword events such as the one shown above in this subsection. Assuming Reach were modified to handle such subword events, its ceiling performance on phosphorylation events would be 92.9% precision and 81.9% recall, for an F1 of 87.1% on the development partition, demonstrating the possibility of a score considerably higher than the one reported by the TEES system (83.9% precision and 83.5% recall). The other FNs were caused by faulty syntactic parsing (10%), misidentifying causes (7%), unhandled errors in the input such as ‘phosphor**ly**ation’ (6%), latent arguments that are only supplied by domain knowledge (3%) and missing rule coverage (16%).

Lastly, please note that this difference in task definitions works both ways: Team 1 in Table 4 trained and tuned their components using the BioNLP data sets. As the table shows, this yielded low throughput compared to Reach in the Big Mechanism evaluation.

## Identification of mutex alterations of cancer drivers

We applied this NLP framework to multiple biological data analysis algorithms. The biological data analysis algorithm we focus on in this work identifies mutex alterations of ‘driver’ mutations in cancer. We observe that across a cohort of cancer patients, some mutations co-occur within the same patient less than expected by random chance. This often happens because these alterations unlock the *same* cancer-driving pathways and the positive selection of one diminishes substantially when the other is present. A simple analogy for this problem is the following: consider a burglar that aims to enter a building to reach valuable property. The burglar may break in either through a window or a door to enter, but likely not both, because one entry point is enough to get inside the building. Across a sufficiently large set of burglary cases, broken windows and doors will overlap less than expected. In other words, ‘one is enough.’

One brute-force, no-prior approach to detect mutex relationships is simply to test all pairs of genes using a hypergeometric test. However, we often see that three or more genes within a same pathway exhibit a mutex pattern. In these cases, the basic approach is less useful, simply because the number of hypotheses increases exponentially as a function of the module size, decreasing statistical power because of corrections for multiple comparisons (Here, a *module* is a group of signaling pathways that impact the same downstream protein.). For larger modules, it also becomes more important to explain why a particular module is mutex mechanistically, as there are more confounding factors.

To address this problem, we previously introduced the Mutex algorithm (5), which combines large-scale omic profiles with prior knowledge of pathway mechanisms. Given a set of omic profiles, Mutex performs a graph search on the prior networks derived from pathway information, testing at each step for a network module that is mutually exclusively altered and can explain, by the merit of the underlying pathway structure, the observed pattern. Prior pathway knowledge improves the accuracy of Mutex by limiting the search space and reducing the loss of statistical power. Such knowledge also provides mechanistic explanations of the observed correlations. However, when these pathways come from human-curated databases such as PCs, recall is low due to the aforementioned database coverage issues. Alternatively, Mutex can operate over a fully connected network to produce a no-prior model. This ability provides a basis to study the trade-offs between no-prior, high-coverage methods that do not provide mechanistic explanations and prior-based, lower-coverage methods that may overlook some key events.

In this work, we evaluate whether we can improve the prior-based approach by expanding the knowledge of prior pathways with information extracted by Reach. We compare the results both with the prior-based approach and the no-prior approach.

**Figure 4:**
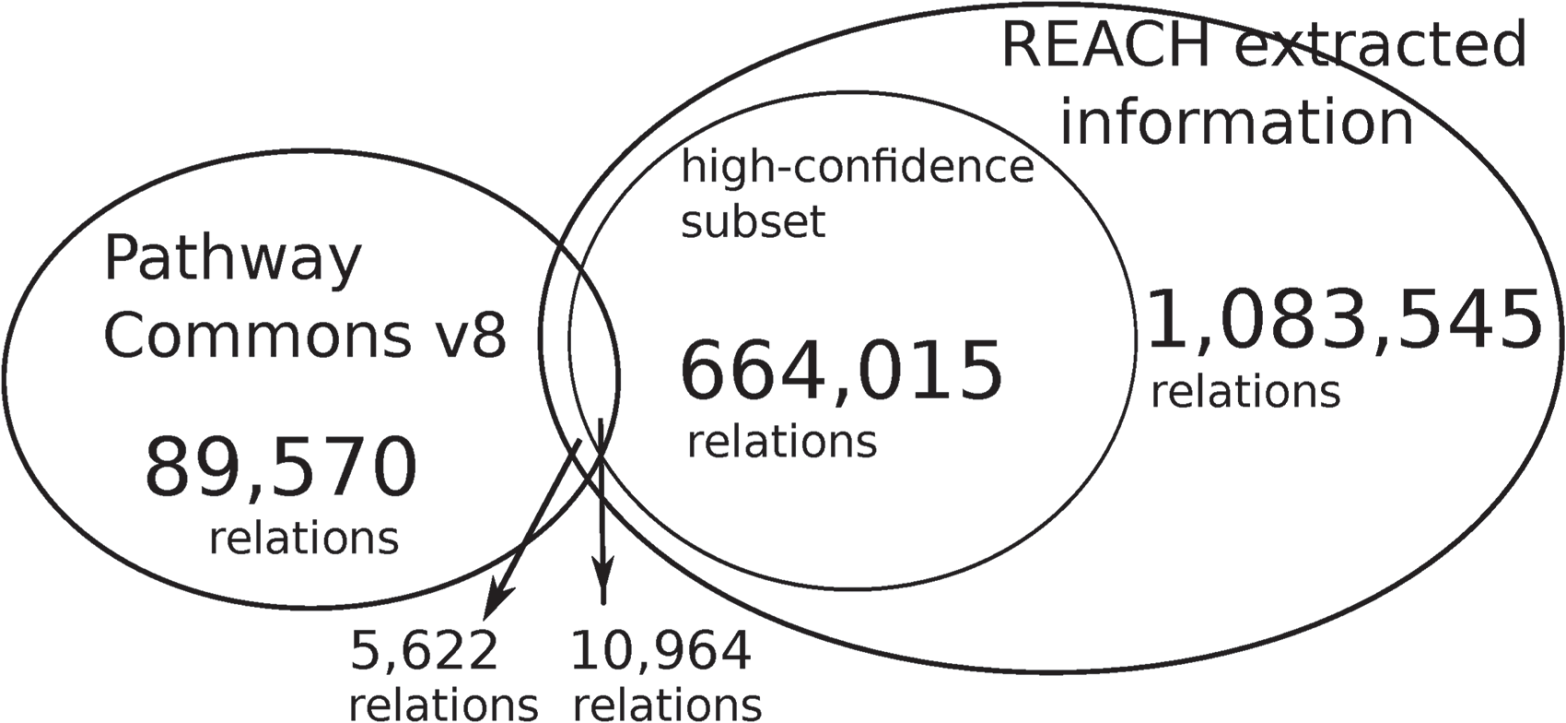
The Reach output is $\sim\! 12$ times larger than the size of PCs. We conjecture that the small overlap is caused by the fact that the Reach interactions are extracted from open-access publications, whereas PCs pathways come mostly from other, paywalled publications. The high-confidence subset is of relations that were found in more than one paper.

**Figure 5:**
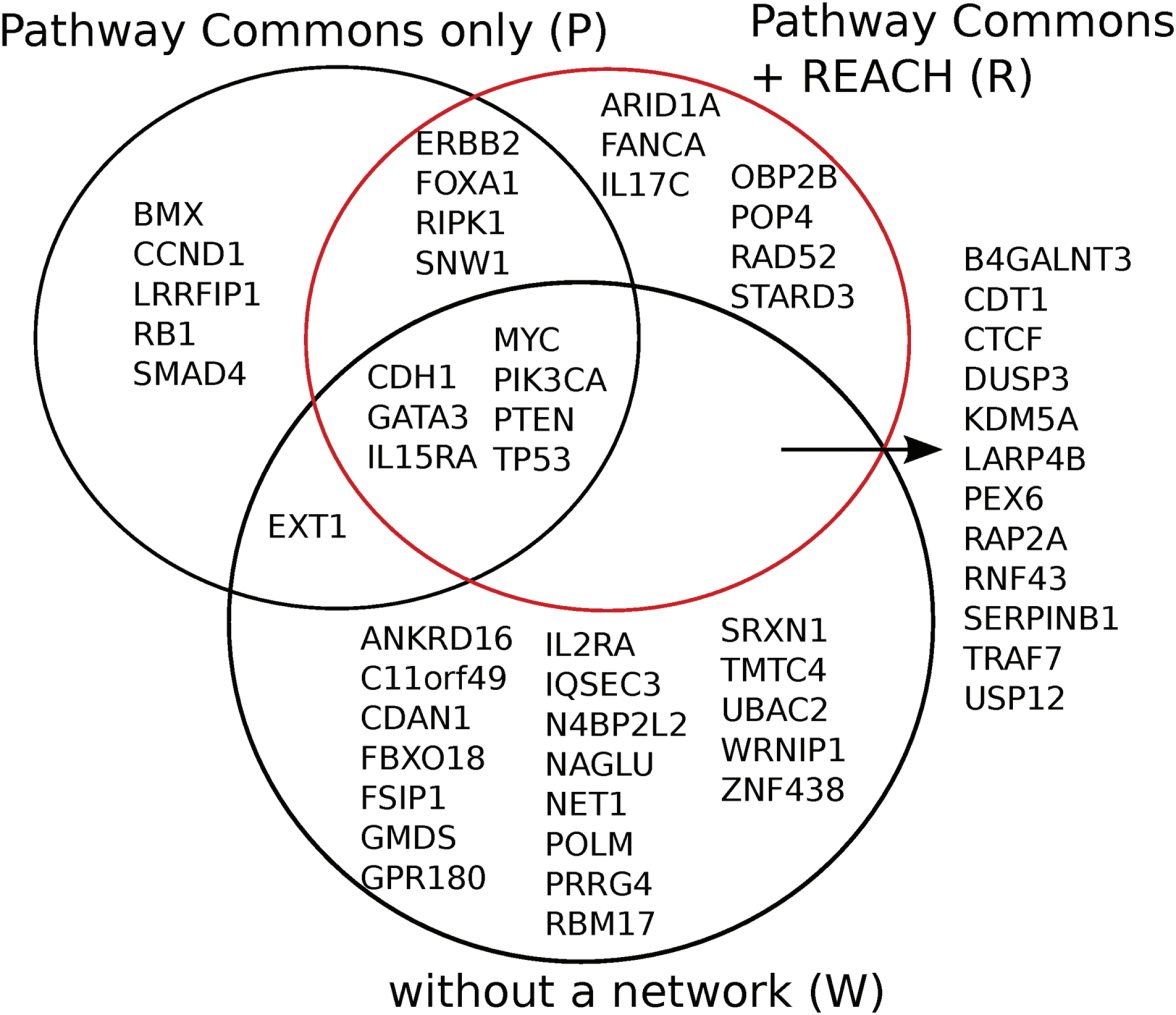
Reach allows Mutex to detect seven new candidate ‘driver’ genes for breast cancer which are not detected otherwise, when using PCs alone, or without using any network. We observed similar results for six other cancers in the TCGA data set.

**Figure 6:**
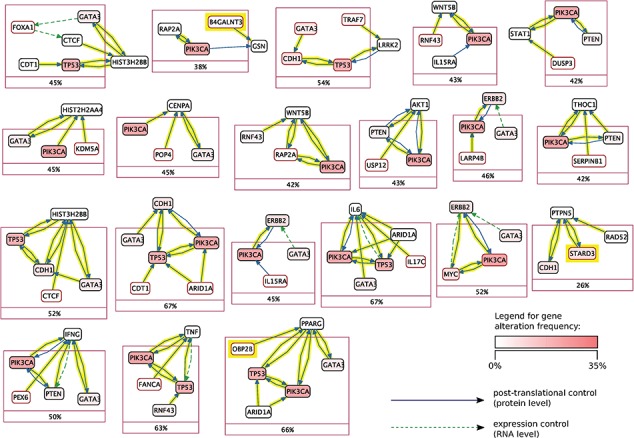
Mutex groups for TCGA breast cancer. This graph shows the interactions of the genes in each Mutex group and their targets. The highlighted relations exist in Reach data but not in PCs. Highlighted genes are not detectable without using Reach data.

## Extrinsic evaluation: discovery of biological hypotheses

This evaluation demonstrates that Reach-extracted pathway fragments improve the inference capacity of the Mutex algorithm, even when it already benefits from large curated models (‘big mechanisms’). Specifically, we extended the PCs (http://www.pathwaycommons.org/) human-curated pathways, which were used by the previously published instance of Mutex, with fragments extracted by Reach from all papers in the Open Access subset of PubMed (1 046 662 papers as of June 2015) (Figure 4).

**Table 5:** Mutex + Reach analysis of TCGA. The **R**−P−W and **R**W−P ablation experiments indicate that Reach extractions are responsible for the discovery of new hypotheses in seven cancers

*Cancer study*	***R***	*P*	*W*	*R-P-W*	*RW-P*
BLCA	2	2	6	0	0
BRCA	30	17	40	7	12
CESC	5	6	7	0	0
DLBC	0	5	0	0	0
GBM	23	14	40	3	7
HNSC	26	23	25	3	2
KICH	0	0	6	0	0
LAML	2	2	2	0	0
LGG	26	12	51	0	14
LIHC	12	17	16	0	0
LUAD	14	16	11	1	0
OV	7	11	7	2	0
PAAD	22	7	17	10	5
SARC	15	22	25	0	0
THCA	9	11	12	0	0
UVM	2	3	34	0	0

Using this combined prior network we were able to identify *previously unidentified* but highly statistically significant mutually exclusively altered signaling modules in TCGA cancer data sets using the Mutex algorithm described above. Figures 5 and 6 show Mutex groups for TCGA breast cancer, and Table 5 summarizes the findings for all enhanced cancer studies in TCGA. ***R*** represents the Mutex configuration using the combined Reach $+$ PCs network, $P$ denotes the Mutex configuration using only the PCs network and $W$ marks the Mutex configuration uninformed by any supporting network. In Table 5 we also include ablation results, e.g. **R**−P−W is the output of the ***R*** configuration without hypotheses discovered by either the $P$ or $W$ approaches. All in all, Table 5 highlights that machine reading is responsible for the discovery of new hypotheses in seven cancers.

**Table 6:** Correctness of the hypotheses generated by Mutex + Reach. The ‘With direction’ column considers strict, directional hypotheses, e.g. *GATA3 activates PTEN*. The ‘Ignoring direction’ column considers non-directional hypotheses, e.g. either *GATA3 activates PTEN* or *PTEN activates GATA3*

	*Hypotheses generated*	*With direction*	*Ignoring direction*
Seen at least once	51	65%	71%
Seen at least twice	21	80%	80%

**Table 7:** Error analysis of the incorrect hypotheses generated by Mutex+Reach

*Error type*	*Frequency*	*Example*	*Incorrect output*
Complex syntax	39% (7)	‘In mouse models of leukemia and melanoma, IDH mutants accelerated cell cycle transition by activation of the MAPK and ERK pathway and repression of tumor suppressors CDKN2A and CDKN2B (Chaturvedi et al., Shibata et al.)’	CDKN2A controls IDH. The correct interaction to be extracted from this statement is: IDH controls CDKN2A.
NER	22% (4)	‘At PND100, BPA significantly increased expression of EGFR ($p = 0.0132$), phospho-IGF-1R ($p = 0.007$), …’	BPA is-a Protein. In this paper, BPA refers to the corresponding chemical not the protein with the same name.
Hedging	22% (4)	‘Therefore, we next investigated whether CIC promotes mutant p53 GOF.’	CIC activates p53, which is unsupported by the hedged statement.
Other	17% (3)	—	

A manual evaluation of these modules by an external cancer researcher (Table 6) reveals that, despite the inherent noise in machine reading, 65% of the hypotheses proposed by the Mutex algorithm that had access to signaling pathways extracted by Reach are indeed correct according to the literature. Further, a simple redundancy filter that keeps Reach extractions only if they are seen at least twice in the literature increased this accuracy to 80%. This demonstrates that our approach systematically and incrementally increases coverage of prior, curated networks using NLP strategies, and, we believe, is valuable for molecular tumor boards and other cases where one needs to combine system-scale data with the knowledge in the literature.

However, a post-hoc error analysis of the incorrect hypotheses proposed by this approach (Table 7) indicates that machine reading is not a solved problem: 39% of the error are generated by incorrect syntactic analyses, 22% by incorrect entity recognition or grounding (e.g. in the example in the table ‘BPA’ refers to the chemical Bysphenol A not the protein with the same name) and 22% are caused by hedged statements that were not supported by experimental results.

### Related work

Reach builds upon the tremendous body of work in language technology applied to bioinformatics that was developed in the past two decades. We summarize the major trends that influenced our work below, but for a more comprehensive background we recommend reviews of the field such as (14).

Because of the above-mentioned information explosion in biomedical research, it is imperative to develop reliable, automated methods to extract information from this literature and make it available in a structured fashion. The BioNLP shared tasks and associated workshops were organized to advance research in this area (22, 23, 35). Many systems have participated in this shared task, broadly representing two directions: rule-based and machine learning methods.

Rule-based information extraction systems have been successful in the biomedical domain. Rule-based systems took off with the advent of Finite State Automaton Text Understanding System (FASTUS) (3), which was implemented as a cascade of finite state automata (FSA), where each FSA captured a ‘layer’ in the task to be addressed (e.g. entities, events), and was defined through a grammar that aggregated multiple rules. Systems such as FASTUS tend to rely on shallow linguistic structure for efficiency. Inspired by the ideas promoted by FASTUS, one of the first rule-based information extraction systems to target the biomedical domain was Blaschke *et al.* (11), which focused on extracting protein–protein interactions. Devised by biologists, the system searches for mentions of proteins separated by a term known to signal their interaction. The extracted protein–protein interactions were then assembled into a small interaction graph with a high degree of accuracy.

While Blaschke *et al.* (11) demonstrated the effectiveness of lexicalized patterns, deeper linguistic analysis affords certain advantages such as better generalization. Kilicoglu and Bergler (20) used a concise set of rules over deep linguistic structure (dependency parses) to detect nine types of biochemical events. This system was one of the top performers in the BioNLP 2009 shared task on event extraction.

An important trend in information extraction is, of course, the use of machine learning. These approaches can be classified in two sub-classes: supervised learning, where the machine learns from data manually annotated by domain experts, and distant supervision, where training data are automatically generated by aligning a database of known facts (e.g. protein–protein interactions) with relevant texts [e.g. biomedical publications discussing such interactions (31)]. The first approach that applied machine learning to biomedical information extraction was proposed by Craven and Kumlien (15). Notably, this is also the first work to use distant supervision for information extraction. Björne *et al.* (10) proposed a supervised machine learning approach for biomedical information extraction, which obtained the best results at the BioNLP 2009 shared task on event extraction. Since then, several efforts have improved upon its performance (13, 30, 33, 34, 44). Notably, the top performers at the more recent editions of the BioNLP shared task rely on machine learning (9, 32).

Reach builds upon this previous work in several ways. First, we propose a declarative rule-based approach that is inspired by and improves upon this body of work, using a framework designed to build grammars that are concise and interpretable and which can mix deep and shallow syntactic analysis. Second, this work addresses additional important phenomena that are generally ignored in previous work (e.g. coreference resolution and event polarity). And third, our approach can be combined with machine learning approaches to discover relevant grammars automatically. Our experiments indicate that such hybrid approaches can be constructed at minimal cost and are successful (41).

We and other groups have previously integrated curated priors into omic analysis and have shown that it improves the accuracy and interpretability of the inferences for a wide range of tasks (1, 4, 6, 12, 17, 26, 45). Of particular note is the DREAM network inference challenge where prior-based methods took the top two positions in an independent evaluation (18). Others have looked at the overlap between curated models and literature-derived networks (25, 37). Our work is the first to carefully examine whether the biochemical pathways extracted by the machine can be successfully combined with human-curated models in the context of a specific analytical task.

## Conclusions

This work showed that the large-scale automated reading of cancer literature ushers in novel cancer research that combines ‘big data’ automatically extracted from the literature with ‘big mechanisms’, i.e. large protein signaling pathways curated by domain experts.

We introduced Reach, a machine reading system that processes statements in the biomedical literature into mechanistic information. An independently administered evaluation demonstrated that the proposed system outperforms other systems under a metric that combines precision and throughput. All in all, Reach achieved a relatively high precision at high throughput, capable of processing one paper in $4.5$ seconds. The system is available as open-source software at github.com/clulab/reach.

We used Reach to process a large number of PubMed Central articles containing mechanistic information and demonstrated that this information improves biological data analysis algorithms. Using a combination of information produced by Reach and PCs, we discovered new cancer-driving mechanisms for seven cancers in the TCGA data set. An external biologist who analyzed the hypotheses proposed by the algorithm found out that 65% of these are correct (i.e. they are supported by the literature). If we consider only interactions seen at least twice in the literature, 80% of the resulting hypotheses are correct.

Beyond the use case discussed in this paper, this approach proposes a pipeline for information analysis in the biomedical domain that we believe generalizes beyond the domain addressed here. In this pipeline, machine reading is used to process a very large number of publications. This has the advantage of scalability beyond human capacity, but the drawback that it introduces noise. To mitigate the latter issue, biological data analysis algorithms (Mutex in this work) filter out the noise by identifying strong associations between machine data and patient data and synthesize the information produced through machine reading into a small number of strong hypotheses. This approach, we believe, is valuable for molecular tumor boards or other cases where one needs to combine system-scale data with the knowledge in the literature.

## Notes

**Database URL:** https://github.com/clulab/reach/wiki/Datasets
